# Supplementary material for: Spatial and temporal trends in western polecat road mortality in Wales
Source: PeerJ. 2022 Dec 1;10:e14291. doi: 10.7717/peerj.14291 (PMC9744138; doi:10.7717/peerj.14291)
Supplement: Supplemental Information 1 — The percentage of total variation explained by each component is given in parentheses. Loadings that explain the largest proportion of each PC are in bold. [file peerj-10-14291-s001.docx]

| Habitat type | Principal Component Axes | | | | |
| --- | --- | --- | --- | --- | --- |
|  | PC4 | PC5 | PC6 | PC7 | PC8 |
| Arable | 0.229 | **-0.656** | 0.356 | **0.359** | -0.210 |
| Broadleaf woodland | -0.268 | -0.016 | -0.364 | **0.600** | -0.152 |
| Coniferous woodland | -0.228 | **-0.533** | 0.146 | -0.384 | -0.332 |
| Heathland | 0.031 | -0.250 | **-0.763** | -0.104 | -0.226 |
| Improved grassland | -0.082 | 0.174 | -0.107 | -0.284 | **-0.615** |
| Semi-natural grassland | 0.284 | 0.310 | 0.151 | **0.424** | **-0.541** |
| Urban | 0.247 | 0.280 | 0.181 | -0.289 | -0.287 |
| Wetland | **-0.821** | 0.135 | 0.267 | 0.095 | -0.137 |
