# Supplementary material for: Spatial and temporal trends in western polecat road mortality in Wales
Source: PeerJ. 2022 Dec 1;10:e14291. doi: 10.7717/peerj.14291 (PMC9744138; doi:10.7717/peerj.14291)
Supplement: Supplemental Information 2 [file peerj-10-14291-s002.docx]

| Name | Model | ΔAIC |
| --- | --- | --- |
| Mod 1 | Rabbit + Elevation + Morans I + Patches + PC1 + PC2 + Road density | 0 |
| Mod 2 | Rabbit + Elevation + Morans + Patches + PC1 + PC2 + Road density + Water density | 0.206 |
| Mod 3 | Rabbit + Elevation + Morans I + Patches + PC1 + Road density | 0.512 |
| Mod 4 | Rabbit + Elevation + Morans I + Patches + PC1 + Road density + Water density | 1.019 |
| Mod 5 | Rabbit + Elevation + Morans I + Patches + PC1 + PC2 + PC3 + Road density | 1.747 |
| Mod 6 | Rabbit + Elevation + Morans I + Patches + PC1 + PC3 + Road density | 1.978 |
